# Network Pharmacology- and Molecular Dynamics Simulation-Based Bioprospection of *Aspalathus linearis* for Type-2 Diabetes Care

**DOI:** 10.3390/metabo12111013

**Published:** 2022-10-24

**Authors:** Ayesha Akoonjee, Athika Rampadarath, Christiana Eleojo Aruwa, Taibat Arinola Ajiboye, Abdulwakeel Ayokun-nun Ajao, Saheed Sabiu

**Affiliations:** 1 Department of Biotechnology and Food Science, Faculty of Applied Sciences, Durban University of Technology, Durban 4000, South Africa; 2 Department of Biochemistry, University of Abuja, Abuja 900105, Nigeria

**Keywords:** rooibos, *Aspalathus linearis*, network pharmacology, molecular dynamics simulation, type 2 diabetes, anti-diabetic activity

## Abstract

The medicinal herb *Aspalathus linearis* (rooibos) is globally recognized in type-2 diabetes mellitus (T2DM) treatment due to its known and distinctive compounds. This work utilized network pharmacology (NP) coupled with molecular dynamics simulation in gaining new insight into the anti-diabetic molecular mechanism of action of rooibos teas. It looked at the interactions between rooibos constituents with various relevant protein receptors and signaling routes associated with T2DM progression. The initial analysis revealed 197 intersecting gene targets and 13 bioactive rooibos constituents linked to T2DM. The interactions between proteins and compounds to the target matrix were generated with the Cystoscope platform and STRING database. These analyses revealed intersecting nodes active in T2DM and hypoxia-inducible factor 1 (HIF-1) as an integral receptors target. In addition, KEGG analysis identified 11 other pathways besides the hub HIF-1 signaling route which may also be targeted in T2DM progression. In final molecular docking and dynamics simulation analysis, a significant binding affinity was confirmed for key compound-protein matrices. As such, the identified rooibos moieties could serve as putative drug candidates for T2DM control and therapy. This study shows rooibos constituents’ interaction with T2DM-linked signaling pathways and target receptors and proposes vitexin, esculin and isovitexin as well as apigenin and kaempferol as respective pharmacologically active rooibos compounds for the modulation of EGFR and IGF1R in the HIF-1 signaling pathway to maintain normal homeostasis and function of the pancreas and pancreatic β-cells in diabetics.

## 1. Introduction

Non-insulin dependent diabetes, also referred to as type 2 diabetes mellitus (T2DM), remains one of the major global causes of mortality and morbidity [1], with nephropathy, cardiovascular diseases, and retinopathy identified as its common secondary complications. The disease affects close to 450 million individuals globally and continues to increase in prevalence [1]. The number of affected persons is, however, predicted to double by the year 2030 [2,3]. The progression of T2DM occurs due to insulin resistance or the impaired secretion of insulin, and the impaired metabolism of proteins, lipids, and carbohydrates [4]. In the latter disease progression stage, there is a major reduction in pancreatic cell function and mass, which invariably affects glucose uptake in adipose tissues, liver and muscle and leads to hyperglycemia [5]. Some risk factors involved in T2DM progression are genetic factors, age, obesity, high blood pressure, inactive lifestyle, smoking, and unacceptable lipid levels [4].

Generally, T2DM can induce an oxidatively stressed condition in the hyperglycemic state [6]. This gives an indication that as an added therapy option, natural bioactive phenol and/or non-phenol compounds with good antioxidant potential from teas could help in T2DM management. Several studies attest that tea consumption decreases the incidence and risk of T2DM [6,7,8]. Teas are the second most consumed non-alcoholic beverages after water [9]. The maceration of roots, stem, flowers, fruits, leaves, seeds, decoction and infusion are ways by which herbal teas such as rooibos are prepared. Interestingly, Africa has a rich biodiversity, with about a quarter of health promoting plants including herbal teas found in the southern Sahara region [10,11]. The rooibos herb is also indigenous to South Africa [12]. Teas with anti-oxidative and anti-diabetic potentials include those from *Cyclopia* sp., *Camelia sinensis* [13,14,15,16,17,18], and rooibos (*Aspalathus linearis*) [12]. In particular, rooibos tea has gained popularity worldwide for its anti-diabetic activity in reducing glucose levels in T2DM management. Rooibos has a rich content of novel and existing bioactive moieties and a distinct flavonoid profile which has been linked to a range of pharmacological activities. Hence, rooibos has great potential for utilization in the medical and pharmaceutical industries [12,19].

The rooibos herbal tea is natural, caffeine-free, and may be consumed in the unfermented (green) or fermented (red) form. Distinct molecules identified only in rooibos herbal tea include asparanin (a cyclic dihydrochalcone) [20] and Aspalathus (a dihydrochalcone glucoside) [21,22,23], both flavonoids. In addition, among the phytochemicals reported in rooibos teas are quercetin-3-*O*-rubinoside, eriodyctiol, isovitexin, vitexin, glucopyranoside, Aspalathus, chrysoberyl, ferulic, caffeic and *p*-coumaric acids, nothofagin, phenylpyruvic, isoquercitrin, orientin, iso-orientin, luteolin-7-*O*-glucopyranoside, rutin, and hyperoside [24], catechin, esculin, lignans, esculetin, and coumarins [25,26]. These constituents inform its great potential for medicinal use in T2DM management and attest to its global popularity [27].

Fermented rooibos tea extracts decreased tissue and plasma malondialdehyde levels, biomarkers associated with liver toxicity in diabetic rats, and advanced glycation end products [28]. In addition, fermented rooibos extracts improved antioxidant enzymes’ activity and inhibited lipid peroxidation in rat liver and plasma [29]. In an ex vivo study, aqueous rooibos extract demonstrated cardio-protective properties on diabetic rats in which an oxidative state and ischemia had been induced [30]. This effect was linked to aspalathin’s ability to prevent hyperglycemia- and high glucose-induced oxidative stress through improved mRNA expression of nuclear factor (erythroid-derived 2)-like 2 (*Nrf2*) and associated antioxidant targets at later stages [31]. Aspalathin in rooibos extracts also demonstrated enhanced glucose metabolism in T2DM and insulin secretion in cultured L6 myotubes [32].

While several studies have set out to determine rooibos tea pharmacological properties and its associated polyphenols using the fermented or unfermented tea extracts or the purified compounds [24,33,34,35,36,37,38,39,40], no data is available using network pharmacology (NP) to determine the molecular bases of rooibos tea against T2DM and the key signaling pathways involved. This approach alongside molecular dynamics (MD) simulation can reveal putative compounds and drug molecules, genetic target, and related signaling pathways linked to specific diseases, and they have been adopted in T2DM studies to predict significant compounds of antidiabetic significance [41,42]. As such, they are useful to speculate bioactive molecules mechanism of action and improve outcomes of future pharmacology-oriented studies [41,42,43]. It is on this background that this study employed NP coupled with MD simulation to give insight into gene targets of interest with a view to further enhance rooibos anti-diabetic applications and guide future studies. The work will also give gene target credence by providing insight into the rooibos mechanisms of action in studies utilizing its related compounds or extracts against T2DM. The study unveils primary targets, potential roles played, and signaling pathways related to rooibos consumption for T2DM management.

## 2. Materials and Methods

### 2.1. Data Mining for Rooibos Bioactive Constituents

The 36 bioactive constituents (Table S1) of rooibos tea employed in this study were obtained from published literature [24,33,34,35,36,37,38,39,40] and Dr. Duke Phytochemical and Ethnobotanical Database (https://phytochem.nal.usda.gov/phytochem/plants/show/2218?et=#act-97821-close (accessed on 4 February 2022)), before being subjected to an initial screening with the Lipinski’s rule of five (Ro5), which is indicative of the drug-likeness attributes of potential oral bioactive molecules/drugs [44]. Subsequently, the identified constituents ADME attributes (Absorption, Distribution, Metabolism and Excretion) were assessed using the Swiss ADME server (http://swissadme.ch/index.php) as reported earlier [19]. Calculations were done using constituent’s SMILES (derived from the PubChem (https://pubchem.ncbi.nlm.nih.gov/ (accessed on 10 July 2022)).

### 2.2. Acquiring and Preparing Identified Compounds and Proteins

The *Homo sapiens* mode was used in acquiring proteins linked to rooibos compound targets by incorporating their ‘SMILES’ into the STP (Swiss Target Prediction) (http://www.swisstargetprediction.ch/) and SEA (Similarity Ensemble Approach) (http://sea.bkslab.org/) databases. Platforms used to collect targets related to T2DM were the DisGeNeT (https://www.disgenet.org/search (accessed on 15 August 2022)) [45], OMIM (https://www.ncbi.nlm.nih.gov/omim) [20] and Malacards (https://www.malacards.org/ (accessed on 15 August 2022)) [21] databases, while the VENNY 2.1 tool (https://bioinfogp.cnb.csic.es/tools/venny/ (accessed on 25 August 2022)) identified separate and overlapping targets between T2DM targets and rooibos bioactive constituents [43].

### 2.3. Network Creation Using Intersecting Targets

In performing protein queries included in the STRING platform (https://string-db.org/ (accessed on 30 August 2022)) were humans within a confidence interval greater than 0.4 in T2DM target genes, interacting proteins, and intersecting targets connecting rooibos compound-associated genes. Thereafter, the Cystoscope program [46] was used to categorize the whole network, followed by the degree algorithm to recognize its integral genes using Equation (1):

Deg (v) = |N(v)|(1)

given that, N(v) = a node neighbor, v = each node’s neighbors.

### 2.4. Layout of Network for Pathway Compound Target (PCT)

A PCT web/matrix was created in a graphical format using the Cystoscope merger algorithm plugin inputted with pre-processed rooibos compounds and T2DM targets which overlapped. A network topology parameter analysis was carried out using the Cystoscope network analyzer, with nodes depicting targets, pathways and compounds. The interactions between node representatives were shown on the edges. The more a node is directly associated with each one, the higher the impact [26].

### 2.5. Assessing Kyoto Encyclopedia of Gens and Genomes (KEGG) Routes in Overlapping Targets

The KEGG pathway interpretation of intersected targets was carried out using the DAVID tool (https://david.ncifcrf.gov/tools.jsp (accessed on 4 September 2022)) to determine the target role in signaling pathways. This is an integral platform for network pharmacology studies since underlying disease molecular mechanisms associated with targets are shown [47]. A *p*-value benchmark of <0.05 was used in pathway enrichment and corrected for with the false discovery rate (FDR) error control method. Results were depicted as ‘Q’ value. The KEGG pathway was visualized with a bubble plot map derived from ‘Microsoft Excel 2022’ for pathways examination.

### 2.6. Ligand and Receptor Preparation for Molecular Docking

The X-ray crystal structures of the key and most significant targets (EGFR (4I23) and IGF1R (3NW6)) identified through KEGG enrichment analysis were obtained from the RSCB Protein Data Bank (https://www.rcsb.org/ (accessed on 10 September 2022)), and prepared by removing water molecules and protein residue connectivity. This was followed by addition of missing side chains using UCSF Chimera software V1.14 [48]. The 3D structures of rooibos compounds associated with EGRF and IGF1R, and the standards (Erlotinib and NVP-ADW742, for EGFR and IGF1R, respectively) were retrieved from PubChem (https://pubchem.ncbi.nlm.nih.gov/ (accessed on 10 September 2022)). These structures were optimized through the addition of Gasteiger charges and non-polar hydrogen atoms [49], and the optimized proteins and compounds were thereafter subjected to molecular docking.

### 2.7. Molecular Docking and Dynamics Simulation Studies

The Autodock Vina Plugin on Chimera was used to dock the optimized compounds into the binding pockets of EGFR and IGF1R. The grid box was defined with a spacing of 1 Å and appropriate sizes pointing in x, y and z directions in each case. The docked complexes with the best pose based on the docking scores were further subjected to MD simulations.

**Figure 1:**
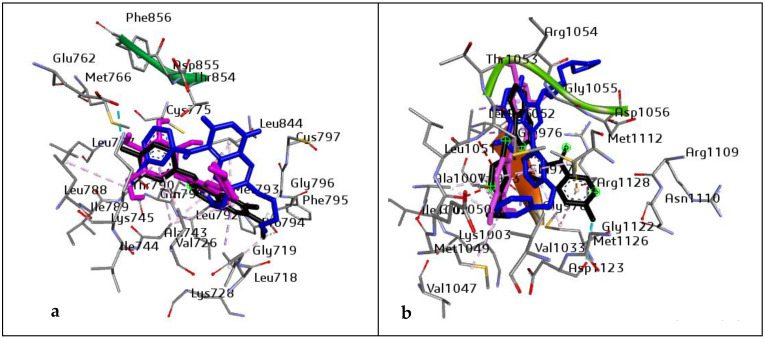
Superimposition on co-crystal structure of (**a**) EGFR (4I23): Black: 4I23 native inhibitor, Blue: standard drug (Erlotinib), and purple: ligand with the highest docking score (Vitexin). RMSD value of 0.6 Å. Grid box coordinates: centre (x = −0.93, y = −54.60, z = −25.4999665218) size (x = 24.4236853013, y = 20.48, z = 26.46); and (**b**) IGF1R (3NW6): Black: 3NW6 native inhibitor, Blue: standard drug (NVP-ADW742), and purple: ligand with the highest docking score (Chrysoeriol). RMSD value of 0.65 Å. Grid box coordinates centre (x = −11.52, y = 2.41, z = 11.78), size (x = 27.105, y = 19.48, z = 18.03).

However, before the MD simulation was executed, the docking protocol was validated to avoid pseudo-positive binding conformations. This was achieved through Root Mean Square Deviation (RMSD) measurement of the docked ligands from the reference pocket bearing the native ligands in the experimental co-crystal structures of EGFR and IGF1R, following optimal superimposition [50]. The RMSD values (0.6 Å and 0.65 Å) obtained between the docked ligands from the native inhibitor in the 3D structures of EGFR and IGF1R, were indicative of the same binding orientation, which ultimately validated the protocol adopted (Figure 1).

The MD simulation was done using the GPU version provided with the AMBER 18 package, in which the FF18SB variant of the AMBER force field was used to describe the systems [50]. The restrained electrostatic potential (RESP) and the general amber force field (GAFF) procedures were used to generate atomic partial charges for the compounds in an ANTECHAMBER. The Leap module of AMBER 18 that was used allowed for the addition of hydrogen atoms Na^+^ and Cl^-^ counter ions for neutralization of all systems [51]. Amino acid residues were numbered, and the system was implicitly suspended in an orthorhombic box of TIP3P water molecules such that all atoms were within 8 Å of any box edge. Solutes were initially minimized using the steepest descent technique followed by 1000 steps of conjugate gradients with an applied restraint potential of 500 kcal/mol. Another 1000 steps were carried out using the conjugate gradient algorithm without restraint. A gradual heating MD simulation from 0–300 K was executed for 50 ps, with systems maintained at fixed volume and number of atoms. The solutes systems were imposed with a potential harmonic restraint of 10 kcal/mol and a collision frequency of 1 ps. Following heating, an equilibration estimating 500 ps of each system was conducted, with temperature kept at 300 K, and pressure at 1 bar, to mimic an isobaric-isothermal ensemble (NPT) [52]. The SHAKE algorithm was used to restrain hydrogen atoms bonds during the MD simulations and was conducted for a period of 60 ns [53]. The step size of each simulation was 2fs, and an SPFP precision model was used. Post-dynamic analysis followed with the analysis of root mean square deviation (RMSD), root means square fluctuation (RMSF), solvent accessible surface area (SASA), and radius of gyration (RoG) using the CPPTRAJ module employed in the AMBER 18 suite. Data plots were thereafter drawn using Origin data analysis software [54]. The free binding energy for each molecular species was calculated using the molecular mechanics with the generalized born surface area method [MM/GBSA] [55].

## 3. Results

### 3.1. Rooibos Constituent Compounds Filtering

Following the screening of 36 rooibos compounds using their drug-likeness attributes, 13 compounds (Table 1) stood out based on adherence to Lipinski’s Ro5. These compounds were classified as key rooibos bioactive molecules for oral application, so far as they did not violate the rule or only violated one attribute of the rule.

### 3.2. Intersecting Compounds’ Targets in the STP and SEA Databases Analyses

**Figure 2:**
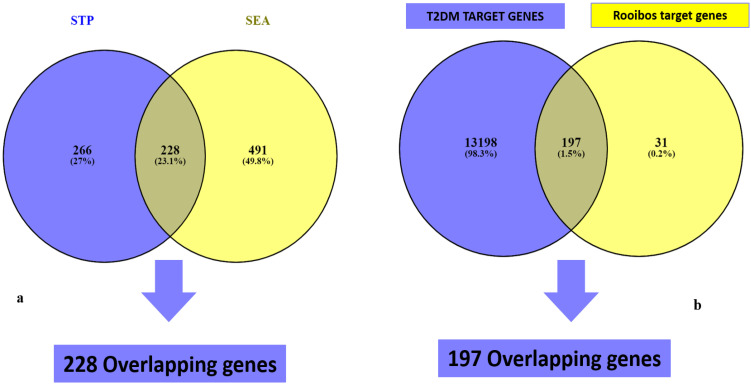
Overlapping genes of rooibos constituents’ related genes from (**a**) SEA and STP, and (**b**) T2DM targets.

Following STP and SEA analysis and duplicates removal, 494 genes associated with STP targets and 719 targets from SEA databases, were detected, respectively. A Venn diagram analysis showed 228 (23.1%) significant overlapping targets between both databases (Figure 2a).

### 3.3. Potential Target Overlaps between T2DM Genes and Rooibos Compounds Linked to 228 Intersecting Genes

The results of the retrieval of T2DM gene targets from related databases revealed the acquisition of 13,395 targets from GeneCards predictions (Figure 2b). Cross-matching of these gene targets with the 228 rooibos compounds-related targets from STP and SEA analysis identified 197 (1.5%) common targets in close and direct association with T2DM and rooibos compounds (Figure 2b). The notable compounds linked to genes potentiating T2DM modulation were the 13 shown in Table 1 and derived from the overlaps between target genes linked to rooibos constituents and T2DM target genes.

### 3.4. Network Analysis (Protein Interactions) of 197 Overlapping Targets

**Figure 3:**
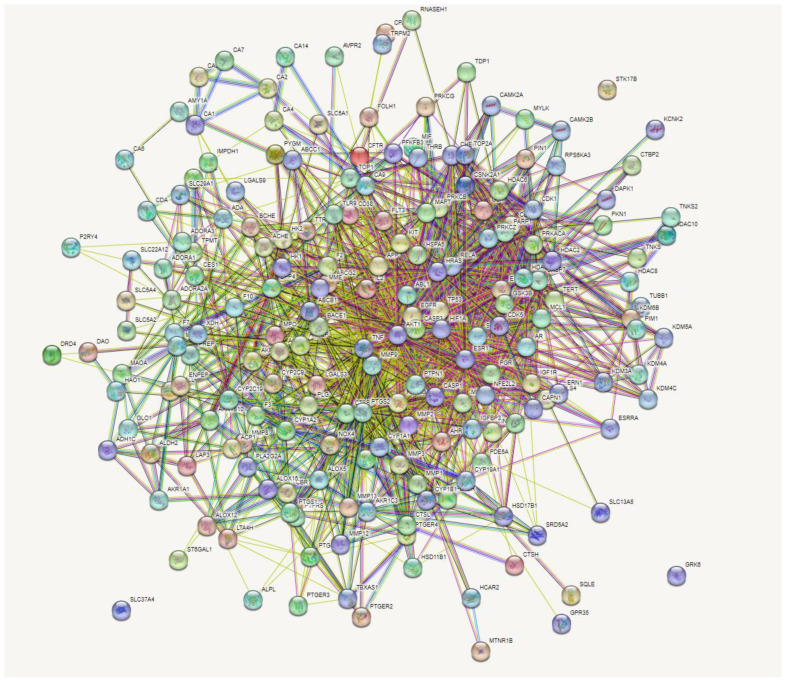
Gene-gene interactions of 197 overlapping genes linked to bioactive constituents of rooibos against T2DM.

The incorporation of the 197 intersecting gene targets into the STRING database generated a significant network among the interacting proteins (Figure 3). In further analysis to determine important central targets within the network, edge numbers linking to respective gene nodes were reported as the number of degrees for each target. Higher degree values determined the leading targets within the network.

### 3.5. Enrichment Pathways (KEGG) Analysis of 197 Overlapping Targets

**Figure 4:**
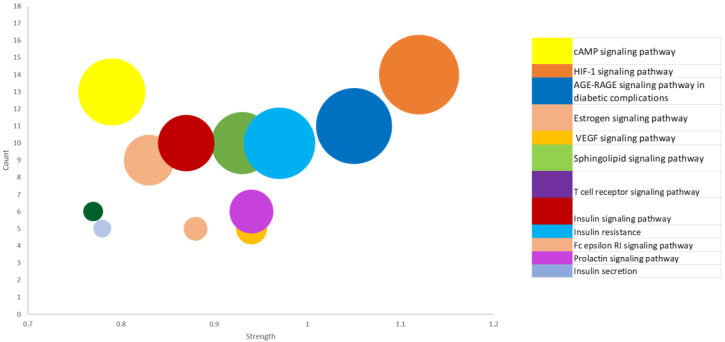
Bubble map showing 12 KEGG enriched signal routes for 197 common targets linked to the occurrence and progression of type-2 diabetes mellitus.

**Figure 5:**
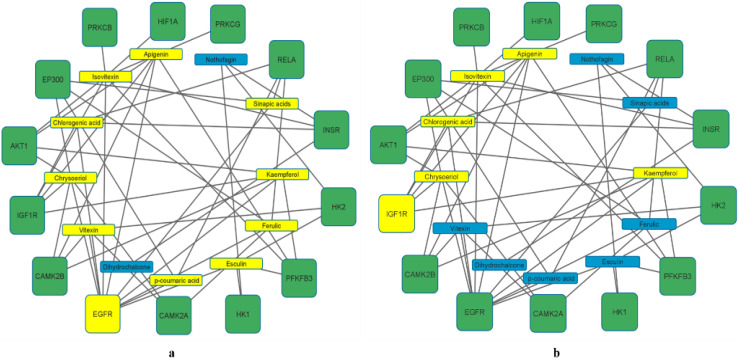
Rooibos compounds (**a**) (10 (in yellow)) showing the highest degree values with EGFR and (**b**) (5 (in yellow)) showing highest degree values with IGF1R gene targets related to the HIF-1 signaling pathway.

**Figure 6:**
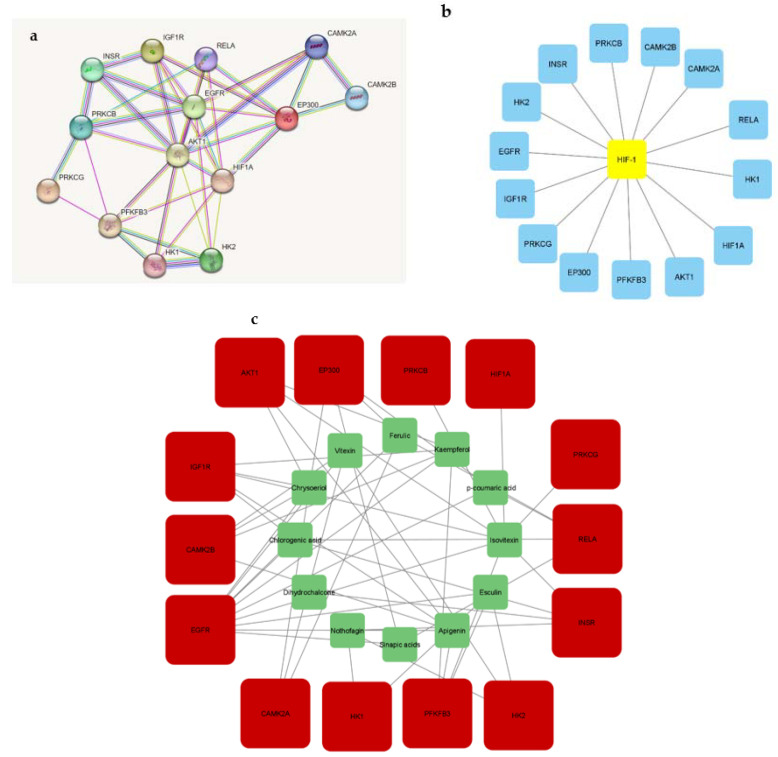
Gene interactions related to (**a**) rooibos compounds; (**b**) T2DM, and (**c**) rooibos compounds linked to HIF-1 pathway genes.

Enrichment of the KEGG pathway was achieved with the DAVID online program on the 197 potential genetic targets and 12 KEGG signaling routes were discovered at a threshold *p*-value of <0.05. Thereafter, the FDR values (or Q values) were calculated which reveal the level of pathway enrichment with a significantly low Q value (Table 2, Figure 4). On further analysis and using the score strength and count from the bubble map generated, the HIF-1 signaling pathway was identified as the best and most enriched route within the assigned intersecting target genes with an FDR value of 7.66 × 10^−10^ (Table 2). Following identification of the key pathway (HIF-1 signaling route) within the network for progression of T2DM disease, of the 14 associated targets in this hub, the EGFR and IGF1R were identified as the hub targets (10- and 5-degree values, respectively) in the HIF-1 key pathway (Table 2, Figure 5). The 10 rooibos compounds linked to the EGFR gene target include vitexin, chrysoberyl, chlorogenic acid, isovitexin, apigenin, sinapic acid, kaempferol, ferulic, esculin, and *p*-courmaric acid (Figure 5a); while those (5) interacting with the IGF1R gene target were chrysoberyl, chlorogenic acid, isovitexin, apigenin, and kaempferol (Figure 5b). Further analysis showed target gene interactions between rooibos compounds (Figure 6a), T2DM targets (Figure 6b) and rooibos compounds associated with the HIF-1 signaling pathway (Figure 6c). Next to the hub targets, NP analysis also showed compound-gene interactions for AKT1, PFKFB3, INSR and CAMK2A targets as the next sets of significant targets in the HIF-1 pathway (Figure S1). The compounds apigenin, isovitexin, chrysoberyl and kaempferol interacted with the AKT1 gene; kaempferol, apigenin, isovitexin and esculin were linked to the PFKFB3 target; is vitexin, nothofagin, chlorogenic acid and dihydrochalcone associated with the INSR target, and vitexin, ferulic acid and chrysoberyl interacted with the CAMK2A genetic target in the HIF-1 signaling route (Figure S1).

### 3.6. Docking Interaction of the Identified Ligands against EGFR and IGF1R in the HIF-1 Signaling Pathway

**Figure 7:**
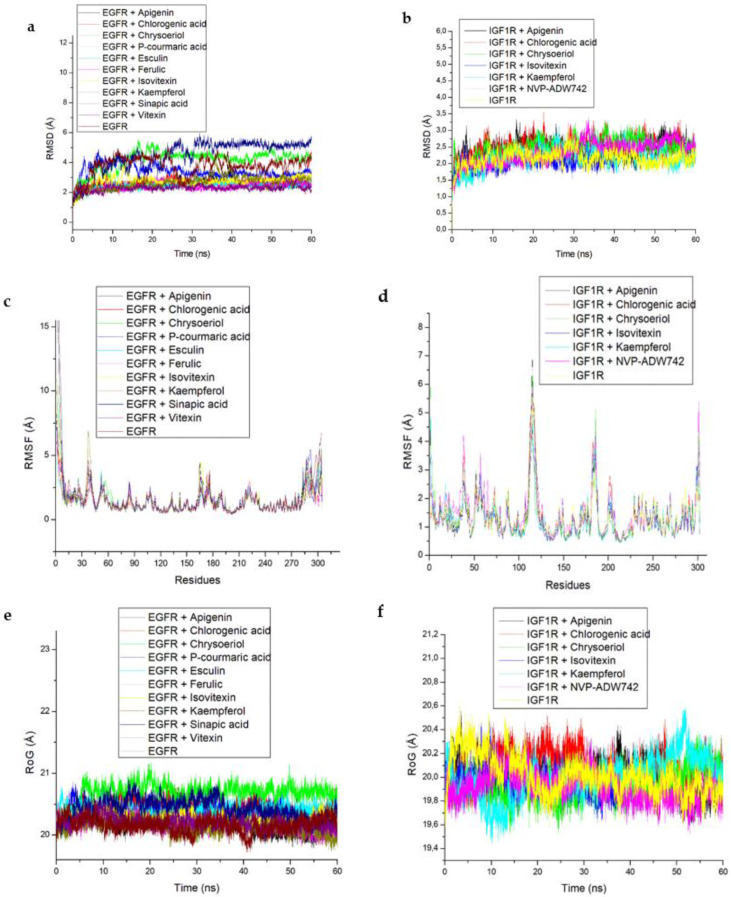

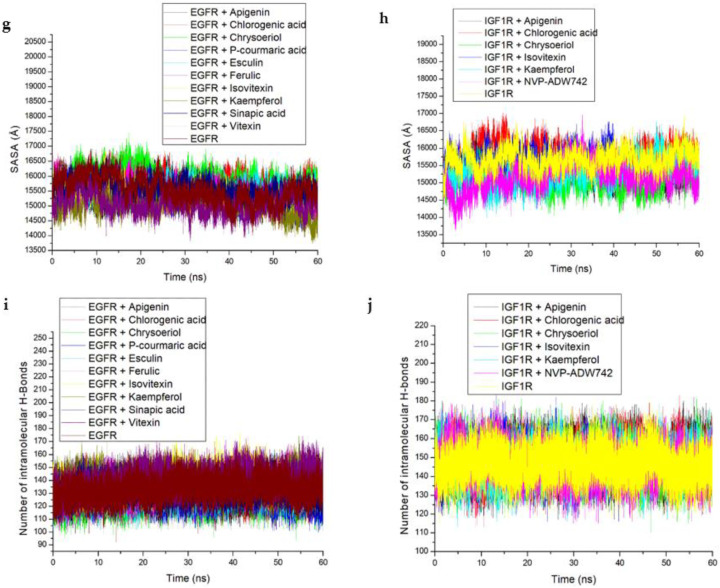
Comparative post-dynamic data showing (**a**,**b**) RMSD, (**c**,**d**) RMSF, (**e**,**f**) RoG, (**g**,**h**) SASA and (**i**,**j**) number of intramolecular H-bond plots of the investigated key rooibos compounds-EGFR and -IGF1R systems over a 60 ns simulation period.

The results of the molecular docking analysis of the identified hub compounds against EGFR and IGF1R are presented in Table 3, with the scores (kcal/mol) following the pattern: vitexin > esculin > chrysoberyl > apigenin > kaempferol for EGFR and higher than the standard (Erlotinib); and chrysoberyl > apigenin > chlorogenic acid > kaempferol > isovitexin for the IGF1R target and higher than the standard (NVP-ADW742). Regarding the thermodynamic energy component analysis, Gibbs free energy binding (ΔG_bind_) of vitexin (−58.61 Kcal/mol) and esculin (−52.43 Kcal/mol) was highest for EGFR, while apigenin (−36.61 Kcal/mol) and kaempferol (−36.44 Kcal/mol) were the highest for the IGF1R (Table 3). The post-molecular dynamics data on the probable structural and conformational changes that could alter the biological activity of the investigated targets are presented as average RoG, RMSF, RMSD and SASA of alpha carbon (Cα) atoms and the number of intramolecular H-bond plots (Table 4 and Figure 7). Here, the vitexin-EGFR bound system had the lowest (2.29 Å) mean RMSD value, and comparatively low average RMSD values were also observed with the AL compound-IGF1R systems (<3.0 Å). Additionally, the RMSD patterns for the EGFR and IGF1R systems reached an initial convergence at 10 ns and 3 ns, respectively, before subsequent divergence at 30 and 8 ns (Figure 7a). It could be observed that the resulting complexes with EGFR and compounds such as chlorogenic acid, esculin, kaempferol, isovitexin and vitexin equilibrated and remained relatively stable after 40 ns and throughout the simulation period (Figure 7a), while similar observations were only noticed with kaempferol and apigenin against IGF1R after 20 ns (Figure 7b). The average RMSF value follows similar patterns as observed with RMSD for the two investigated systems with the vitexin-EGFR complex having the least value (1.40 Å) and generally lower mean values for the IGF1R-bound systems except for the relatively higher values for the NVP-ADW742-complex (1.50 Å) (Table 4). Compared with the IGF1R-bound systems, the RMSF of the EGFR complexes were generally more stable and fluctuated less, and without the prominent vibration observed between residues 100–125 and 175–190 for the IGF1R systems (Figure 7c,d). The observation with RoG revealed similar patterns and comparable mean values for both EGFR and IGF1R systems (Table 4, Figure 7e,f). Similar to the RMSD and RMSF patterns, the vitexin-EGFR system (15,057 Å) had the lowest average SASA value, followed by chrysoberyl- (15,081 Å), kaempferol- (15,276 Å) and apigenin-IGF1R (15,399 Å), relative to the unbound EGFR (15,551 Å) and IGF1R (15,672 Å), respectively (Table 4). Furthermore, it was observed that both the EGFR and IGF1R systems had consistent fluctuation patterns regarding SASA throughout the simulation time (Figure 7g,h). Furthermore, stable fluctuation patterns were seen in the number of hydrogen bonds formed and varied between 100–160 and 125–160 for the EGFR and IGF1R systems, respectively (Figure 7i,j). Notably, of the EGFR systems, the vitexin-EGFR complex had formed the highest number of H-bonds during the simulation period, while that of the IGF1R system ranged between 146.79 for both the unbound species and NVP-ADW742 complex to 148.95 for chrysoberyl (Table 4).

**Figure 8:**
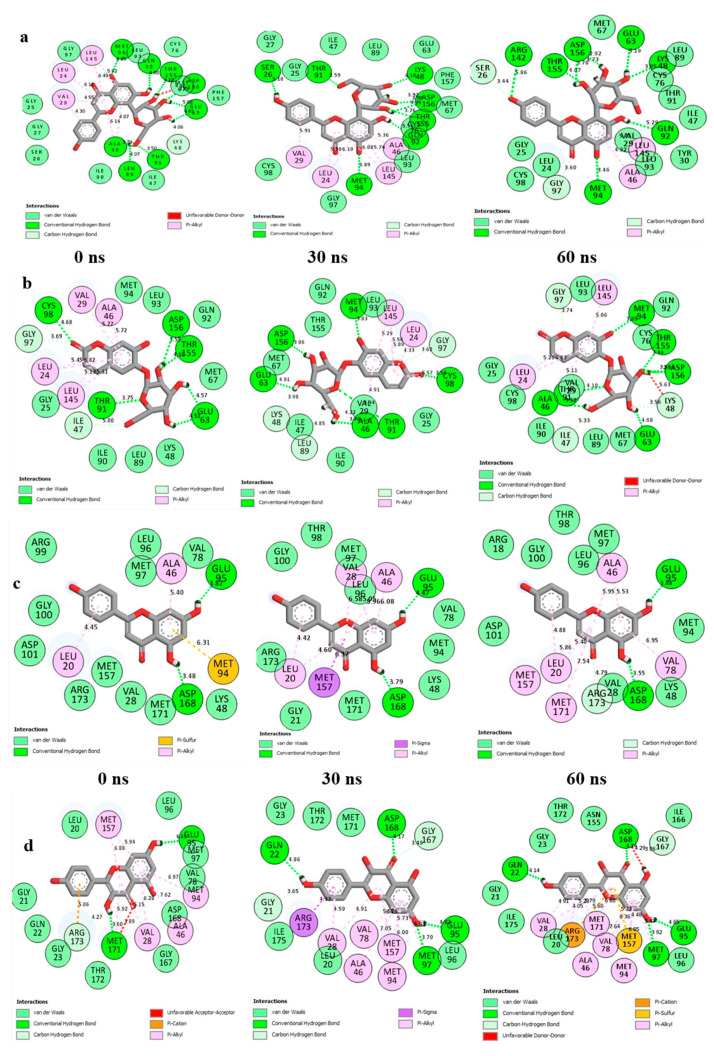
2D interaction plots of (**a**) EGFR with Vitexin; (**b**) EGFR with Esculin; (**c**) IGF1R with Apigenin, and (**d**) IGF1R with Kaempferol, over a 60 ns simulation period.

The data obtained regarding the interaction plots of the investigated compounds against EGFR and IGF1R revealed diverse bond types such as hydrogen (conventional, carbon and π-donor), attractive charge, van der Waals, amide π-stacked, π-sigma, π-cation, π-alkyl, alky, π- sulphur, unfavourable acceptor–acceptor and donor-donor interactions (Figure 8, Table S2). Specifically, the binding of vitexin with EGFR was achieved via seven H-bonds with ARG142, THR155, ASP156, LYS48, GLN92, MET94 and GLU63 (Figure 8a), while esculin-EGFR had six H-bonds (THR155, ASP156, MET94, GLU63, ALA46 and THR91) (Figure 8b). On the other hand, apigenin-IGF1R established two H-bonds with GLU95 and ASP168 (Figure 8c), compared to kaempferol-IGF1R via four H-bonds (ASP168, MET97, GLN22 and GLU95) (Figure 8d), and chrysoberyl with IGF1R via four H-bonds with GLN22, LEU20, MET97 and GLU95 amino acid residues (Table S2), which were conserved over the entire simulation period. Besides the common van der Waals interactions with GLY25 and ILE47 as well as LEU96 established with vitexin/esculin-EGFR and apigenin/kaempferol-IGF1R systems, respectively, conserved π-alkyl bonds formed with LEU146 and ALA46 were also observed throughout the simulation period for the vitexin/esculin-EGFR and apigenin/kaempferol-IGF1R complexes, respectively (Figure 8).

## 4. Discussion

Determining the therapeutic efficacy or mechanism of action of plant fractions is a complicated process, especially for each drug-target, since extracts contain more than one chemical species [56]. On the other hand, specific plant constituent-gene target network system analysis comes in handy with great advantages in revealing new information on the efficiency and mechanism of action of medicinal herbs/plant extracts and their individual pure components. The utilization of computational network pharmacology (NP) techniques has unveiled novel drug candidates or compounds, genetic target profiles, and connected signaling pathways associated with various infectious and non-infectious diseases. The ability of NP to hypothesize the mechanism behind plant fractions synergism and compounds’ activities is key to appropriately redirect future research interest for enhanced pharmacological outputs [43,57].

Within the scope of this work, rooibos constituents were harnessed from various natural compounds’ literature and databases and key constituents’ targets were profiled through the analysis of target intercepts linked to T2DM targets to unearth molecular mechanistic pathways of rooibos (*Aspalathus linearis* (AL)) in T2DM care. This was essential because, although widely known in Africa and globally, the herb’s precise anti-diabetic molecular mechanism of action was not clearly elucidated. We therefore set out to predict the molecular mechanism of action of AL compounds and their interaction with T2DM genes and pathways.

The pathway compound target (PCT) network showed 197 T2DM gene targets associated with 13 of the 36 rooibos constituents screened. A rooibos constituents-gene targets network analysis specified that the bioactive efficacy of AL against T2DM is linked to several genes and signaling pathways. The assessment of the KEGG enrichment path unearthed 12 pathways, 10 of which were signaling pathways and the others were linked to insulin secretion and resistance that were in direct association with T2DM development and progression. This finding indicates that the identified signaling routes could be integral in the action of *Aspalathus linearis* (AL) against T2DM. The relationships between the various determined T2DM related pathways are herein discussed.

The HIF-1 signaling pathway results in diabetes and complications which include the major breakdown of HIF-1α protein and reduced oxygen levels in cells and tissues [58]. The cAMP signaling pathway is also involved in glucose regulation and balance, as well as glucose catabolism, glycogen synthesis, glucagon and insulin production and gluconeogenesis [59], while the sphingolipid signaling pathway is essential to lipids signaling and has a well-known function in diabetes mellitus and insulin resistance pathogenesis and progress [60]. The VEGF pathway, on the other hand, is closely associated with diabetes development, and is usually detected in up-regulated levels in individuals suffering from T2DM [61]. Prolactin signaling pathway: Higher levels of prolactin indicates increased disposition or risk of developing T2DM, while normal amounts point to a reduced risk. Thus, this pathway is important in stalling DM development [62]. Estrogen signaling pathway: Study has shown that estrogen is linked to lowered T2DM risk human cells and postmenopausal mice models. This route targets specific gut and pancreatic cells to enhance glucose tolerance [63]. Fc epsilon RI signaling pathway: This pathway has been shown to enhance the effect of insulin in mice bone marrow [64]. T cell receptor activation: The presence of hyperactive T cells in DM individuals may indicate loss of innate modulatory mechanism. As such, T cells involvement in T2DM may reduce T cell receptor stimulation [65]. The AGE-RAGE signaling pathway in diabetic complications: AGE-(RAGE) receptor activation constitutes a major pathological response to T2DM. Hence, AGE-(RAGE) drug agonists may function as good therapy options in T2DM care [66]. Insulin signaling pathway: This pathway modulates insulin receptor signals and insulin secretion, and maintains glucose balance by regulating glucose uptake or synthesis in tissues and cells, and is influenced by several factors. Again, when insulin signaling is impaired it results in insulin resistance. Thus, dysregulation of insulin control can result perturbed energy and sugar homeostasis with consequential T2DM development [67]. With respect to metabolic pathways linked to insulin secretion and resistance, T2DM-related metabolic symptoms that develop due to inflammation often result in insulin resistance and stressed cells. Deficiency in insulin secretion also adversely affects certain metabolic pathways, leading to hyperglycemia [68]. Insulin resistance is also favorably linked to up-regulated amounts of calcium ions (Ca^2+^) in human serum [69]. Green rooibos fractions have been shown to reduce inflammation signal pathways and overturn induced insulin resistance by obstructing palmitate-mediated nuclear factor-kappa B (NF-KB) activation in adipocyte cells and tissues [70]. Rooibos extracts also possess demonstrable potential to ameliorate insulin resistance in hepatic cells via the enhancement of sensitivity to insulin [71].

Furthermore, the strength or rich factor is used to define the number of relevant genes assigned to a pathway [72]. As such, the higher the strength, the greater the degree of enrichment (DEG). The HIF-1 signal route had the highest DEG of all signaling pathways identified. As a result, this work mainly expatiated on the mechanism of action linked to the hub metabolic pathway and gene (HIF-1). Nevertheless, the other identified gene targets with lower DEGs might also be good putative targets to explore. Overall, the central mechanism of anti-diabetic action of AL extracts and compounds in T2DM care could be linked to maintenance of sugar (glucose) balance through HIF-1 signaling pathway receptors activation, as observed in this study.

The highest docking scores of vitexin and chrysoberyl could be indicative of a better orientation at the binding sites of EGFR and IGF1R, respectively. Still, molecular docking only provides data for predicting compound fitness at a protein active site. As such, there is more reliance on binding conformation data revealed from evaluating compound to protein target systems using calculated binding energy values and MD simulation [73]. The binding free energy (∆G_bind_) also gives an indication of the level of protein inhibition and may be linked to structural/conformational changes which may influence the target’s biological activities [50]. Hence, the highest negative ∆G_bind_ indicative of significant binding affinity recorded for vitexin and esculin, as well as apigenin and kaempferol following the 60 ns simulation period in this study, could be an indication of better interactions with EGFR and IGF1R, respectively. In addition, post-dynamics analysis of bound and unbound complex systems showed lower RMSD (<3.5 Å) for complexes formed from compound-EGFR and compound-IGF1R interactions and indicated better structural complex stability. The RMSD measures the degree of convergence, stability or deviations produced by a protein in a simulation system with lower values suggestive of remarkable/significant stability of the resulting complex [74]. More specifically, the lowest mean RMSD value observed with vitexin-EGFR complex in this study does not only justify its higher negative ∆G_bind_ value but also attests to the high affinity of vitexin towards EGFR. The observation of RMSD towards complexes formed with IGF1R also indicated that all the test compounds including apigenin and kaempferol did not distort the stability of IGF1R judging by the lower values (<3.5 Å) obtained for each resulting system and agrees with a previous study [54], where unaffected structural stability of the resulting complexes was attributed to lower average RMSD values of the investigated systems. The RMSF value indicates the effect the binding compound has on active site residue behavior, with lower or higher alpha (α)-carbon (C) shifts indicating less or more flexible movements, respectively [75]. The RMSF following a similar pattern as the RMSD in this study could be an indication of better flexibility and lesser fluctuation with little or no distortion, which is suggestive of remarkable stability of the complexes, especially for the vitexin-EGFR and apigenin-IGF1R systems. Furthermore, the increased fluctuations in the RMSF of all the IGF1R systems at residues 100–125 and 175–190 could be an indication of the enhanced potential of the test compounds to adapt perfectly to the binding pocket of IGF1R, which subsequently promotes the stability of the resulting complexes. On the other hand, the RoG measures molecule-target complex systems overall structural flexibility and compactness, such that induced changes from compound binding a target could influence its biological property [76]. The relatively similar and insignificant variations in the mean RoG values observed in this study for both IGF1R and EGFR could mean that binding of the test compounds to the two targets do not compromise the stability and compactness of alpha-carbon of the resulting complexes. Unlike RoG, the measurement of SASA shows the surface area of formed complexes accessibility to solvents and the effect of compound-target systems on SASA, with a high SASA value showing increased surface area and decreased system stability, and vice versa [77,78]. In this study, the lower and comparable mean SASA values of the bound complexes (vitexin-EGFR, and apigenin-, and kaempferol-IGF1R) relative to the unbound system is not only indicative of lesser protein residue to solvent molecule interactions and increased systems stability, but is also consistent with the ∆G_bind_ values and further attest to the affinity of vitexin as well as apigenin and kaempferol for EGFR and IGF1R, respectively. These observations with RMSF, RoG and SASA in this study agrees with previous findings [56,76] on ligand-protein interactions for possible drug candidates’ identification and development.

The high number of intramolecular H-bonds for vitexin-EGFR (140.17), and 147.04–148.95 range for rooibos compounds-IGF1R interactions also depict greater intra-structural stability of the resulting complex in each case. This observation suggests the occupancy of some intramolecular space by the vitexin against EGFR and the other test compounds against IGF1R and this observed increase in the intramolecular H-bonds during the 60 ns simulation supports the resulting H-bond interactions between these compounds and the respective targets and is also consistent with other post-dynamic data and particularly the ∆G_bind_ values for vitexin-EGFR and apigenin-IGF1R complexes.

Again, the major bonds in the interaction plots observed in this study post-MD simulation include the pi-sigma, pi-sulfur, pi-alkyl, unfavorable donor-donor, van der Waals and pi-cation interactions, which possess reduced strength in comparison to the observed hydrogen bonds [both C-H and conventional hydrogen (H)]. Intramolecular hydrogen bonds make up one of the strongest and most essential bonds in the discovery of compounds with pharmacological attributes [79,80,81]. This could be evidently seen with the vitexin-EGFR, esculin-EGFR, apigenin-IGF1R, and kaempferol-IGF1R complexes in this study. Other significant interactions like van der Waals and π-alkyl also contributed towards the stability of the investigated complexes. It is noteworthy that the presence of repulsive unfavorable bonds (with exception of vitexin and apigenin post-simulation) and higher bond lengths may also be responsible for the reduced binding free energies reported for esculin and kaempferol, as binding free energies of compound-target systems are known to be influenced by the nature, length and number of interacting bonds [50,82].

Considering the foregoing, the highest binding free energy of the vitexin-EGFR and apigenin-IGF1R systems in HIF-1 activation could mean that these hub compounds have key functions in AL anti-diabetic activity. These findings suggest that these key rooibos constituents may be responsible for the anti-diabetic activity reported for AL tea products and could be further exploited for pharmaceutical anti-diabetic drugs development. The results are also in alignment with the thermodynamic profiles where the best binding free energy correlates with better compactness and structural stability of bound target complexes.

## 5. Conclusions

Within the scope of this research, we proposed the molecular mechanisms of action of rooibos tea associated bioactive compounds for T2DM management using network pharmacology and MD simulation. The findings demonstrated that rooibos tea constituents, especially vitexin, esculin and isovitexin, as well as apigenin and kaempferol, may elicit their respective anti-diabetic functions by modulating two key targets (EGFR and IGF1R) of the HIF-1 signaling pathway, as further validated by MD simulation. Aside from this, the 12 T2DM-related signaling pathway was identified alongside the 13 most active rooibos constituents which significantly affected the key HIF-1 pathway in T2DM regulation. Hence, it is proposed that the identified compounds are pharmacologically active in rooibos tea and could modulate the activation of HIF-1 genes of the HIF-1 signaling pathway implicated in T2DM. This could mean that these rooibos constituents would enhance HIF-1 mediated responses in a way that could maintain the normal homeostasis and function of the pancreas and pancreatic β-cells as well as the prevention of teratogenicity and its attendant secondary complications in diabetics. Overall, this study provided verifiable first-hand findings to buttress the use of rooibos tea in T2DM therapy, while highlighting significant insights into the bioactive molecules, putative overlapping of the genetic target, and mechanism of action of rooibos tea products against T2DM as opposed to previous studies [28,31,32,70,71] that evaluated rooibos tea and its constituents in different experimental models of diabetes, without prior knowledge of the specific pathways or targets of interest to be profiled. This study therefore adds significant findings to the current body of scientific knowledge regarding the use of rooibos tea constituents as T2DM therapeutics and recommends further validation in vitro and in vivo.

## Figures and Tables

**Table 1:** The 13 rooibos compounds that passed Lipinski’s rule.

S/N	Compounds	Passed Lipinski’s Rule/ No. of Violations
1	(+)-catechin	Yes
2	Apigenin	Yes
3	Chlorogenic acid	Yes/1
4	Chrysoeriol	Yes
5	Dihydrochalcone	Yes
6	Esculin	Yes
7	Ferulic	Yes
8	Isovitexin	Yes
9	Kaempferol	Yes
10	Nothofagin	Yes/1
11	*p*-coumaric acid	Yes
12	Sinapic acid	Yes
13	Vitexin	Yes/1

**Table 2:** Target genes in KEGG signaling pathways enrichment related to T2DM.

Pathway	No of Genes	Score	FDR	Genes
AGE-RAGE signaling pathway in diabetic complications	11	1.05	2.20 × 10^−7^	MMP2, NOX4, PRKCB, CASP3, F3, PIM1, PRKCZ, RELA, TNF, HRAS, AKT1
Prolactin signalling pathway	6	0.94	0.00036	GSK3B, ESR2, RELA, ESR1, HRAS, AKT1
Estrogen signalling pathway	9	0.83	6.57 × 10^−5^	MMP2, EGFR, PRKACA, PGR, ESR2, MMP9, ESR1, HRAS, AKT1
Fc epsilon RI signalling pathway	5	0.88	0.0018	ALOX5, SYK, TNF, HRAS, AKT1
T cell receptor signaling pathway	6	0.77	0.0018	IL2, GSK3B, RELA, TNF, HRAS, AKT1
VEGF signalling pathway	5	0.94	0.0011	PRKCG, PRKCB, PTGS2, HRAS, AKT1
Sphingolipid signalling pathway	10	0.93	4.95 × 10^−6^	PRKCG, TP53, PRKCB, ADORA1, PRKCZ, ABCC1, RELA, TNF, HRAS, AKT1
Insulin signalling pathway	10	0.87	1.25 × 10^−5^	PYGM, HK2, INSR, PRKACA, GSK3B, PTPN1, PRKCZ, HK1, HRAS, AKT1
Insulin secretion	5	0.78	0.0041	PRKCG, PRKCB, PRKACA, CAMK2B, CAMK2A
Insulin resistance	10	0.97	3.16 × 10^−6^	PYGM, INSR, PRKCB, GSK3B, PTPN1, PRKCZ, RPS6KA3, RELA, TNF, AKT1
cAMP signalling pathway	13	0.79	3.62 × 10^−6^	CFTR, GLI1, PTGER2, EP300, PRKACA, ADORA2A, PTGER3, ADORA1, HCAR2, CAMK2B, CAMK2A, RELA, AKT1
HIF-1 signalling pathway	14	1.12	7.66 × 10^−10^	EP300, PRKCG, IGF1R, EGFR, HK2, INSR, PRKCB, CAMK2B, CAMK2A, RELA, HK1, HIF1A, PFKFB3, AKT1

FDR: False discovery rate.

**Table 3:** Docking score (kcal/mol) and thermodynamic energy components of key rooibos compounds and targets in the HIF-1 pathway.

	Energy Components (Kcal/mol)					
Complex	Docking Score	ΔE_vdW_	ΔE_elec_	ΔG_gas_	ΔG_solv_	ΔG_bind_
**EGFR**						
Apigenin	−8.4	−32.1 ± 3.71	−23.23 ± 12.10	−55.72 ± 11.10	22.04 ± 7.30	−33.68 ± 6.21
Chlorogenic acid	−7.5	−36.77 ± 4.19	−58.50 ± 10.07	−95.28 ± 8.35	53.69 ± 6.70	−41.59 ± 4.30
Chrysoeriol	−8.6	−41.49 ± 2.93	−14.12 ± 9.73	−55.62 ± 9.28	18.88 ± 6.95	−36.74 ± 4.15
Esculin	−9.4	−43.75 ± 3.75	−67.08 ± 9.15	−110.83 ± 8.28	58.40 ± 6.00	−52.43 ± 4.69
Ferulic	−6.1	−25.88 ± 2.81	−28.24 ± 6.00	−54.13 ± 6.06	28.28 ± 4.87	−25.84 ± 3.48
Isovitexin	−7.0	−38.40 ± 4.59	−60.38 ± 14.16	−98.78 ± 12.60	55.55 ± 8.72	−43.23 ± 5.78
Kaempferol	−8.4	−38.61 ± 3.28	−37.73 ± 9.76	−76.34 ± 9.39	37.66 ± 6.22	−38.68 ± 4.78
*P*−courmaric acid	−5.5	−19.90 ± 2.84	−26.83 ± 5.51	−46.73 ± 5.35	15.43 ± 3.07	−31.30 ± 3.93
Sinapic acid	−6.3	−29.46 ± 2.51	−21.08 ± 9.69	−50.54 ± 9.93	25.26 ± 6.19	−25.27± 4.49
Vitexin	−9.8	−44.85 ± 4.01	−68.30 ± 12.61	−113.15 ± 11.48	54.53 ± 7.06	−58.61 ± 6.67
Erlotinib	−7.2	−	−	−	−	−
**IGF1R**						
Apigenin	−7.7	−33.39 ± 2.71	−27.10 ± 6.10	−60.49 ± 5.33	23.88 ± 3.43	−36.61 ± 2.97
Chlorogenic acid	−7.3	−33.36 ± 4.85	−24.14 ± 20.17	−57.50 ± 17.77	27.74 ± 11.79	−29.75 ± 7.41
Chrysoeriol	−7.9	−36.78 ± 2.49	−14.82 ± 6.38	−51.60 ± 6.10	18.71 ± 4.80	−32.89 ± 2.88
Isovitexin	−6.8	−31.31 ± 4.76	−26.05 ± 9.96	−57.36 ± 9.83	29.99 ± 6.29	−27.36 ± 4.82
Kaempferol	−7.1	−37.69 ± 3.12	−24.94 ± 6.05	−62.64 ± 6.37	26.20 ± 3.58	−36.44 ± 3.80
NVP−ADW742	−7.4	−42.94 ± 3.73	−180.05 ± 13.38	−223.00 ± 15.12	187.28 ± 12.97	−35.71 ± 4.50

ΔE_vdW_: van der Waals energy; ΔE_elec_: electrostatic energy; ΔE_gas_: gas-phase free energy; ΔG_solv_ solvation free energy; ΔG_bind_: total binding free energy; -: Not determined.

**Table 4:** Post-molecular dynamics parameters of key rooibos compounds’ interactions with hub targets in the HIF-1 pathway.

Post-Dynamics Data					
Complex	RMSD (Å)	RMSF (Å)	RoG (Å)	SASA (Å)	Number of H-Bonds
**EGFR**					
Unbound	3.78 ± 0.62	1.76 ± 1.83	20.16 ± 0.13	15551 ± 431	133.42 ± 820
Apigenin	2.52 ± 0.3	1.49 ± 0.95	20.19 ± 0.13	15495 ± 359	132.58 ± 7.96
Chlorogenic acid	2.66 ± 0.31	1.41 ± 0.93	20.42 ± 0.15	15800 ± 305	130.85 ± 7.56
Chrysoeriol	4.19 ± 0.64	1.62 ± 1.30	20.68 ± 0.15	15915 ± 375	127.25 ± 7.81
Esculin	2.44 ± 0.21	1.43 ± 0.84	20.39 ± 0.09	15536 ± 273	131.77 ± 7.76
Ferulic	2.40 ± 0.24	1.46 ± 0.84	20.18 ± 0.10	15430 ± 340	134.90 ± 8.39
Isovitexin	2.75 ± 0.35	1.43 ± 0.96	20.26 ± 0.09	15383 ± 322	138.34 ± 8.57
Kaempferol	2.64 ± 0.43	1.51 ± 1.17	20.14 ± 0.11	15103 ± 374	138.18 ± 7.99
*P*-courmaric acid	3.42 ± 0.48	1.48 ± 0.92	20.35 ± 0.11	15395 ± 363	129.04 ± 7.84
Sinapic acid	4.51 ± 0.95	1.80 ± 2.18	20.43 ± 0.15	15569 ± 295	132.93 ± 7.74
Vitexin	2.29 ± 0.20	1.40 ± 0.94	20.19 ± 0.09	15057 ± 329	140.17 ± 8.82
**IGF1R**					
Unbound	2.16 ± 0.23	1.38 ± 0.72	20. 01 ± 0.15	15672 ± 286	146.79 ± 7.91
Apigenin	2.41 ± 0.27	1.31 ± 0.84	20.02 ± 0.12	15399 ± 283	148.65 ± 8.05
Chlorogenic acid	2.49 ± 0.27	1.36 ± 0.73	20.07 ± 0.13	15787 ± 336	148.70 ± 8.06
Chrysoeriol	2.47 ± 0.30	1.30 ± 0.81	19.94 ± 0.11	15081 ± 294	147.95 ± 7.92
Isovitexin	2.01 ± 0.19	1.29 ± 0.70	19.97 ± 0.10	15673 ± 288	148.45 ± 7.95
Kaempferol	2.16 ± 0.32	1.31 ± 0.73	19.99 ± 0.16	15276 ± 361	147.04 ± 7.92
NVP-ADW742	2.33 ± 0.33	1.50 ± 0,82	19.91 ± 0.11	15167 ± 393	146.79 ± 7.91

## Data Availability

The data are contained within the article or Supplementary Materials.

## Supplementary Materials

The following supporting information can be downloaded at: https://www.mdpi.com/article/10.3390/metabo12111013/s1, Figure S1: Rooibos compounds (4, 4, 4 and 3, respectively) with good interactions linked to other genes (AKT1, PFKFB3, INSR and CAMPK2A) related to the HIF-1 signaling pathway; Table S1: Complete list of rooibos compounds investigated and details of Lipinski’s violation; Table S2: 2D interactions of key targets and their associated key rooibos compounds in the HIF-1 pathway.
