# Supplementary material for: Network Pharmacology- and Molecular Dynamics Simulation-Based Bioprospection of Aspalathus linearis for Type-2 Diabetes Care
Source: Metabolites. 2022 Oct 24;12(11):1013. doi: 10.3390/metabo12111013 (PMC9692680; doi:10.3390/metabo12111013)
Supplement: Supplementary file 1 [file metabolites-12-01013-s001.zip › metabolites-1976397-supplementary.pdf]

Supplementary data

Table S1. Complete list of rooibos compounds investigated and details of Lipinski's violation

S/N	Compounds	Passed Lipinski's/ no of violations	The 13 compounds
1	(+)-catechin	Yes	**1**
2	Apigenin	Yes	**2**
3	Aspalathin	No - 2	
4	Carlinoside	No - 3	
5	Chlorogenic acid	Yes - 1	**3**
6	Chrysoeriol	Yes	**4**
7	Dihydrochalcone	Yes	**5**
8	Eriodictyol 5,3′di-O-glucoside	No - 3	
9	Esculin	Yes	**6**
10	Ferulic	Yes	**7**
11	Hyperoside	No - 2	
12	Isocarlinoside	No - 3	
13	Isoorientin	No - 2	
14	Isoquercitrin	No - 2	
15	Isovitexin	Yes	**8**
16	Kaempferol	Yes	**9**
17	Luteolin	No - 2	
18	Luteolin-7-O-glucoside	No - 2	
19	Neocarlinoside	No - 3	
20	Nothofagin	Yes - 1	**10**
21	Orientin	No - 2 violations	
22	Patuletin 7-glucoside	No - 2 violations	
23	*p*-coumaric acid	Yes	**11**
24	Procyanidin B3	No - 3	
25	Quercetin	No	
26	Quercetin-3-*O*-arabinoglucoside	No - 3	
27	Quercetin-3-*O*-galactoside	No - 2	
28	Quercetin-3-*O*-glucoside	No - 2	
29	Quercetin-3-*O*-robinobioside	No - 3	
30	Quercitrin	No - 2	
31	Rutin	No - 3	
32	Safflomin	No - 3	
33	Scoparin	No - 2	
34	Sinapic acid	Yes	**12**
35	Vicenin-2	No	
36	Vitexin	Yes - 1	**13**

Table S2. 2D interactions of key targets and their associated key rooibos compounds in the HIF-1 pathway.

**Compounds**		**2D interactions**
**EGFR**		
Apigenin	60 ns	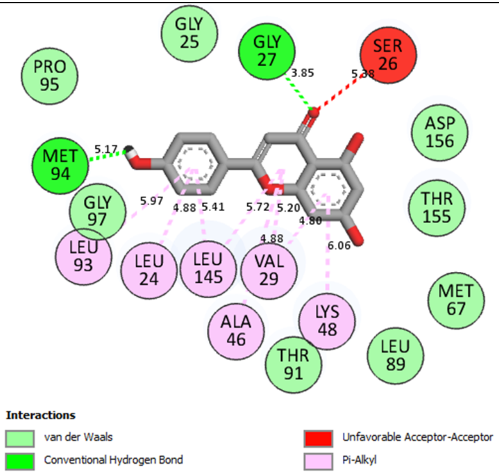
	30 ns	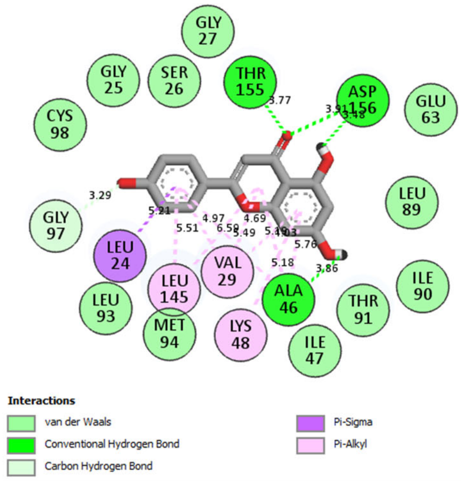
	0 ns	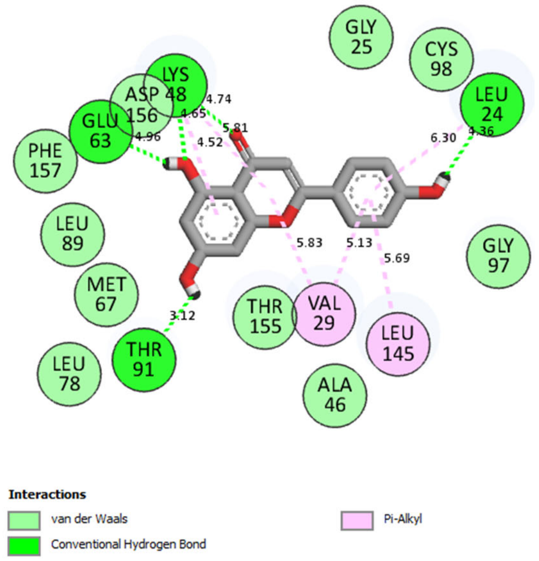

Chlorogenic acid

60 ns

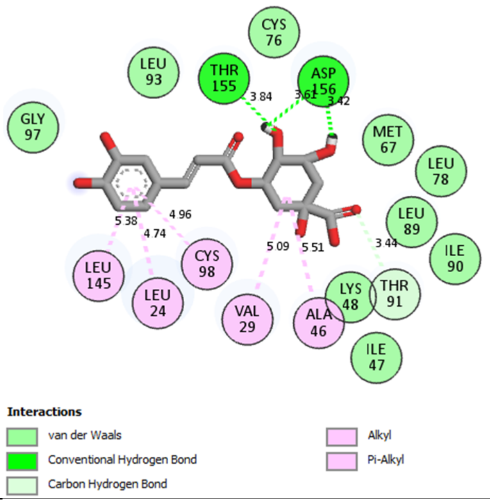

30 ns

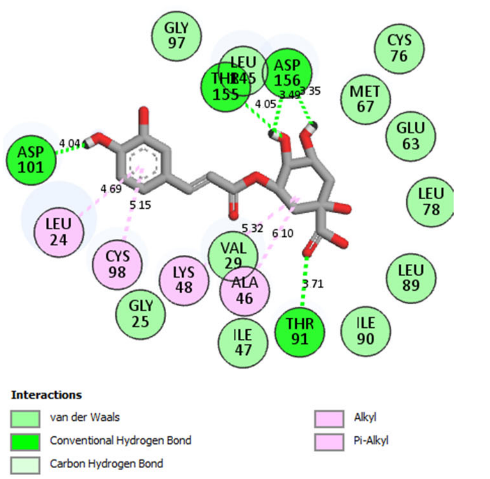

0 ns

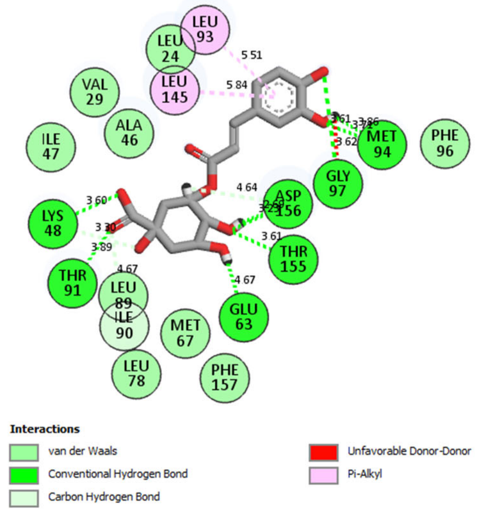

Chrysoeriol

60 ns

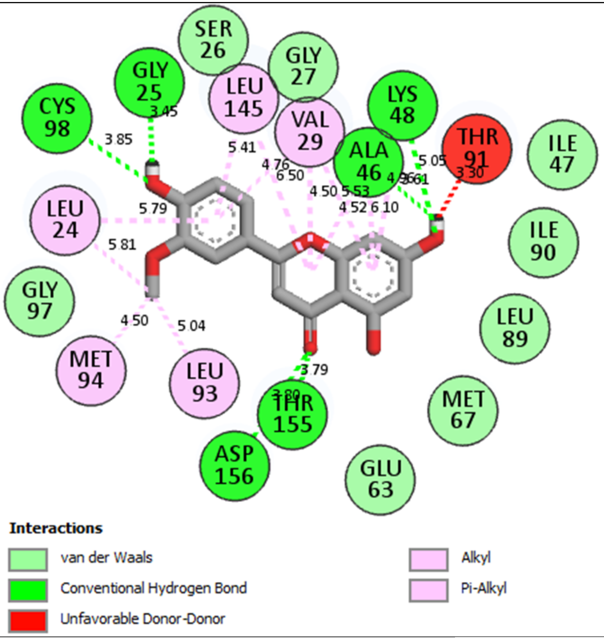

30 ns

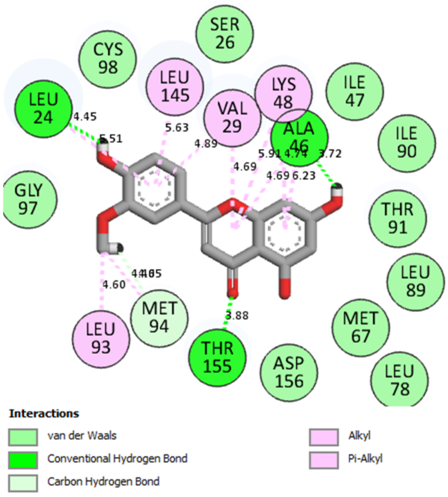

0 ns

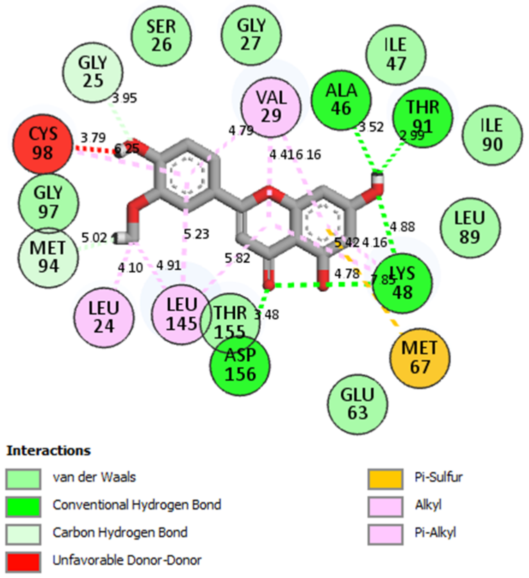

Ferulic

60 ns

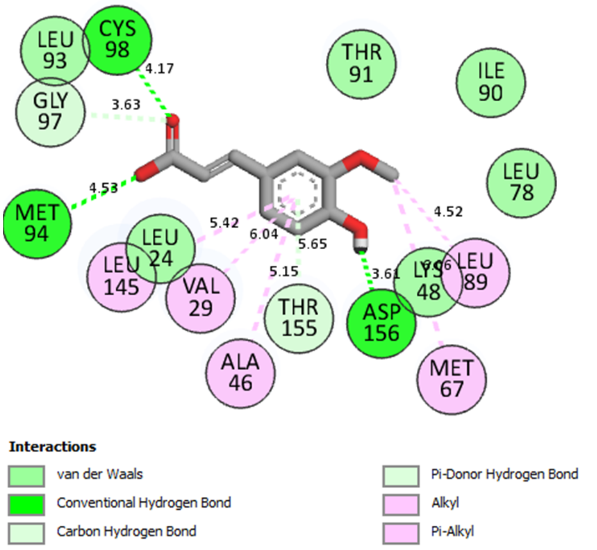

30 ns

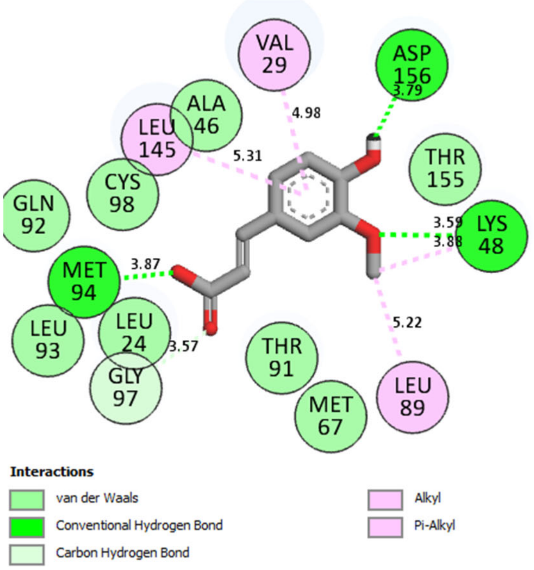

0 ns

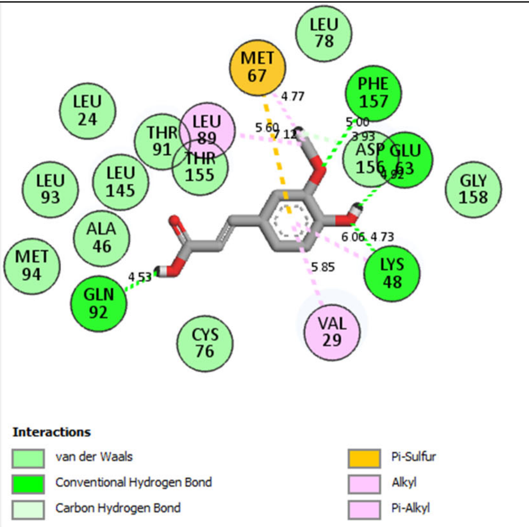

Isovitexin	60 ns	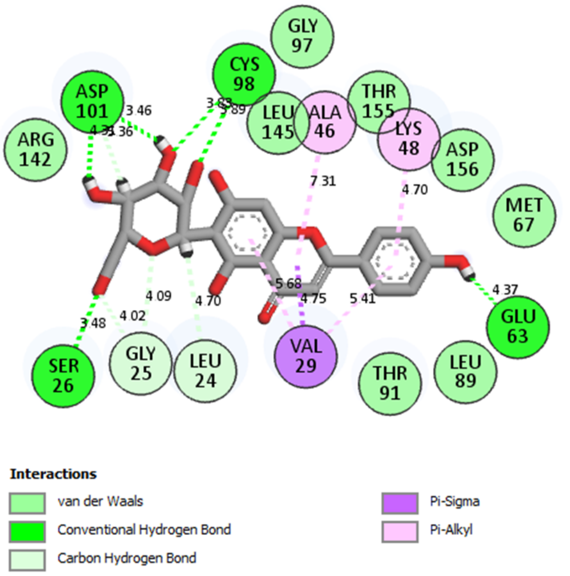
	30 ns	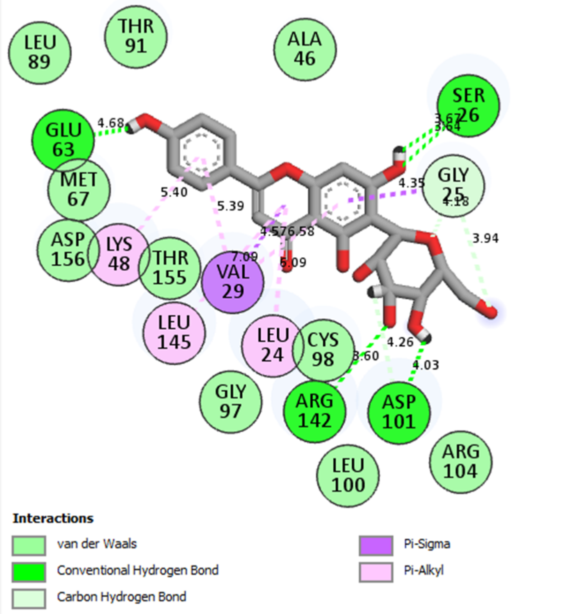
	0 ns	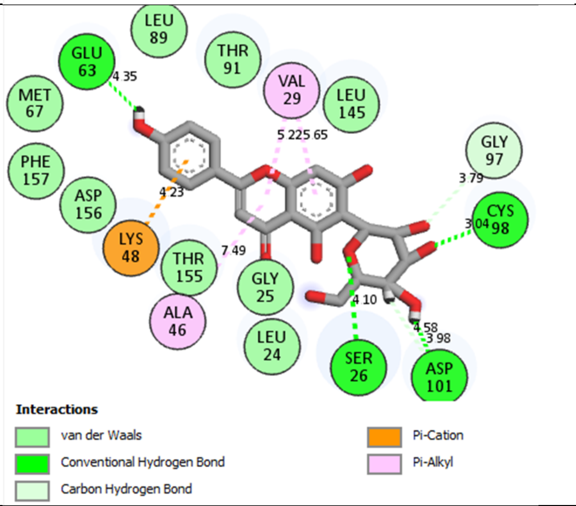

Kaempferol 60 ns

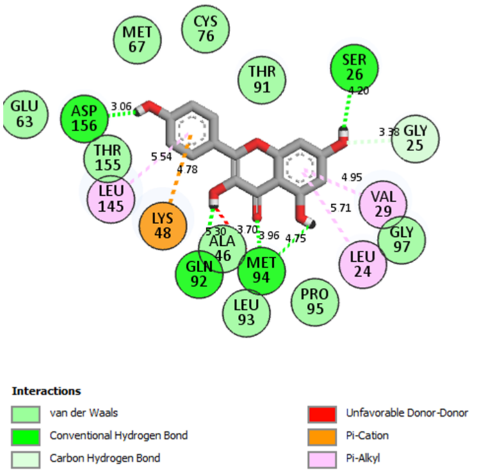

30 ns

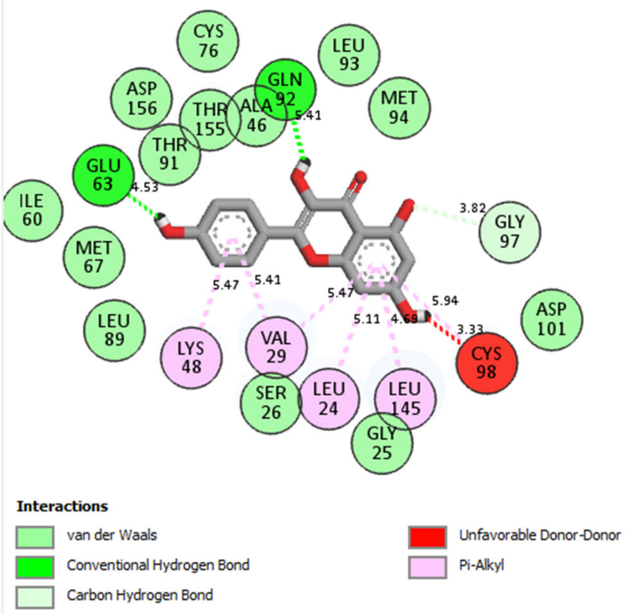

0 ns

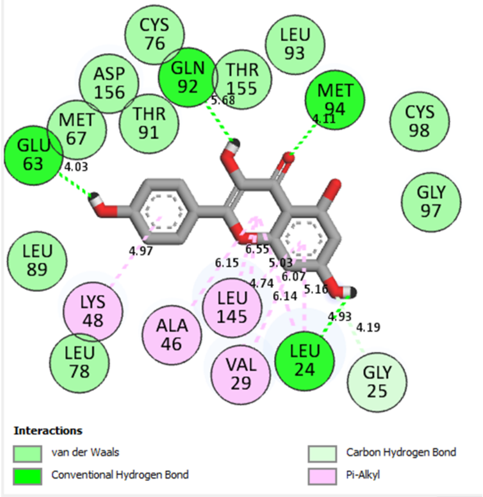

<table>
<tr>
<td>P-courmaric acid</td>
<td>60 ns</td>
<td>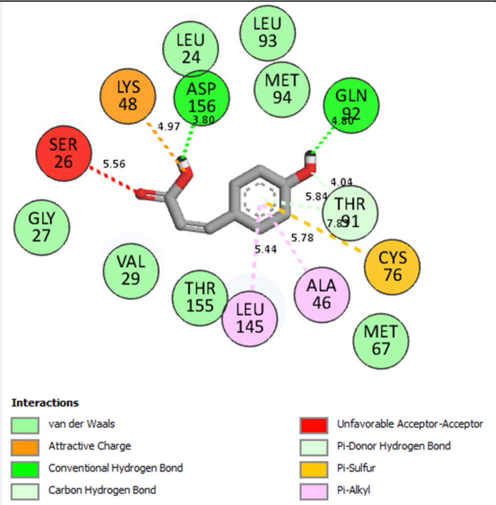
</td>
</tr>
<tr>
<td></td>
<td>30 ns</td>
<td>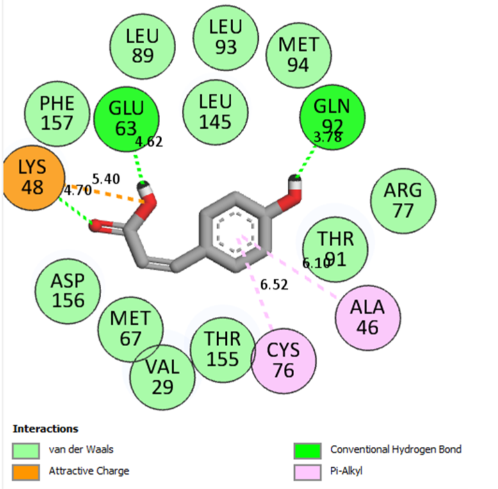
</td>
</tr>
<tr>
<td></td>
<td>0 ns</td>
<td>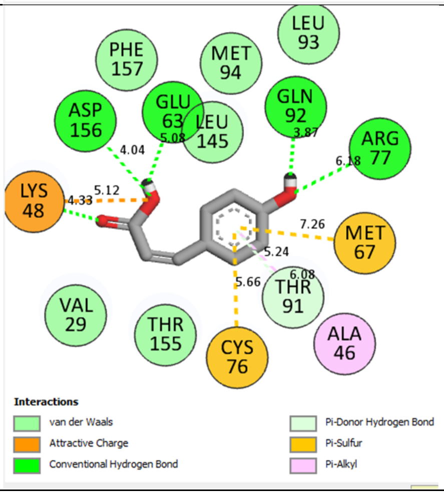
</td>
</tr>
</table>

<table>
<tr><td>Sinapic acid</td><td>60 ns</td><td>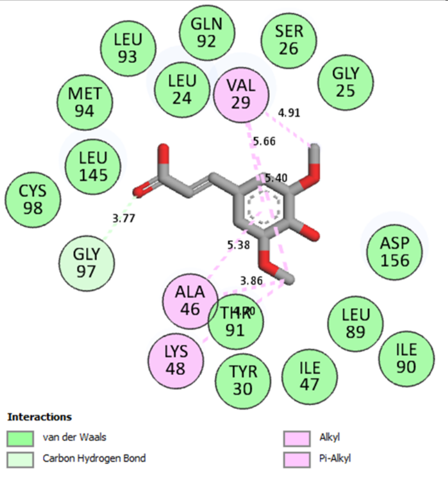
</td></tr>
<tr><td></td><td>30 ns</td><td>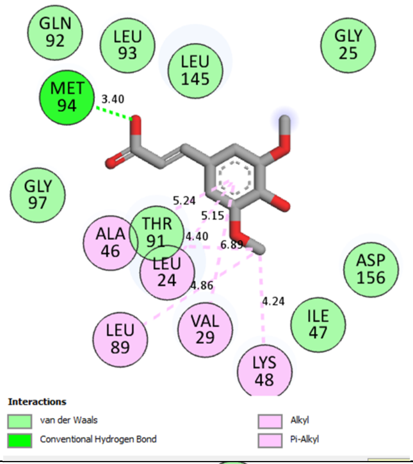
</td></tr>
<tr><td></td><td>0 ns</td><td>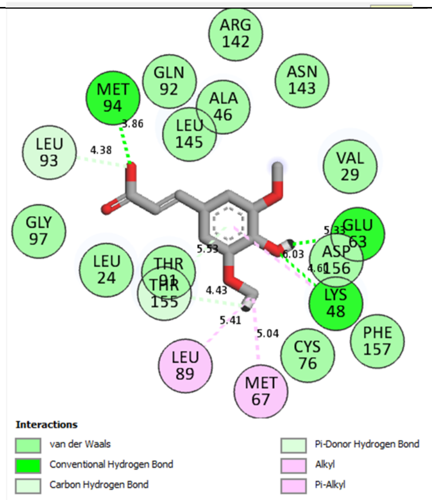
</td></tr>
<tr><td>IGF1R</td><td></td><td></td></tr>
</table>

<table>
<tr><td>Chlorogenic acid</td><td></td><td>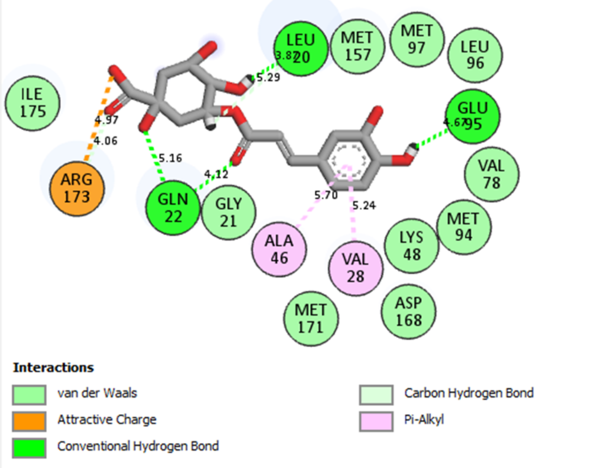
</td></tr>
<tr><td></td><td>30 ns</td><td>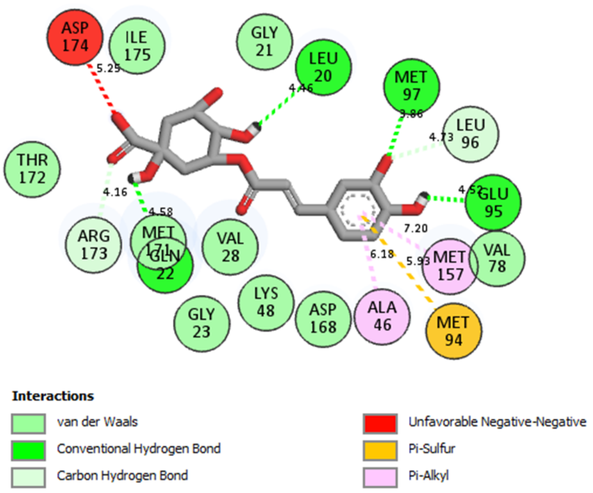
</td></tr>
<tr><td></td><td>0 ns</td><td>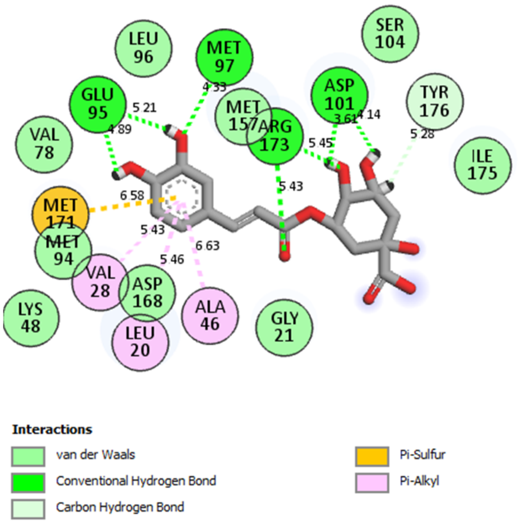
</td></tr>
</table>

Chrysoeriol

60 ns

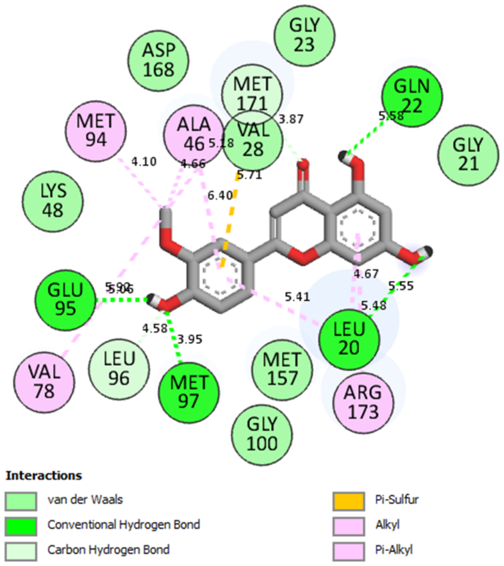

30 ns

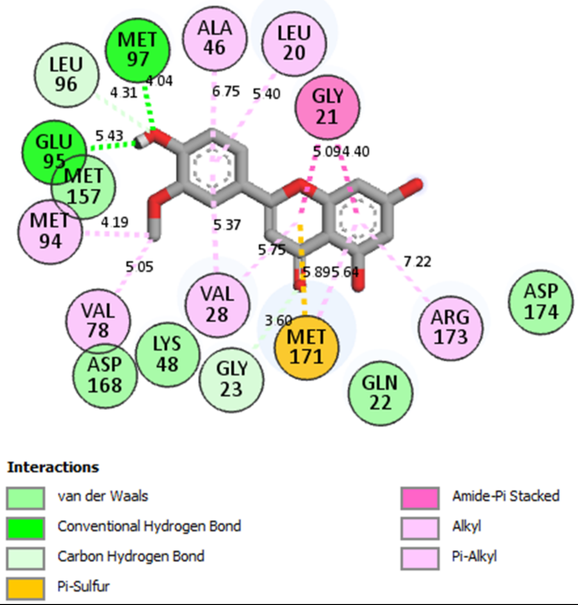

0 ns

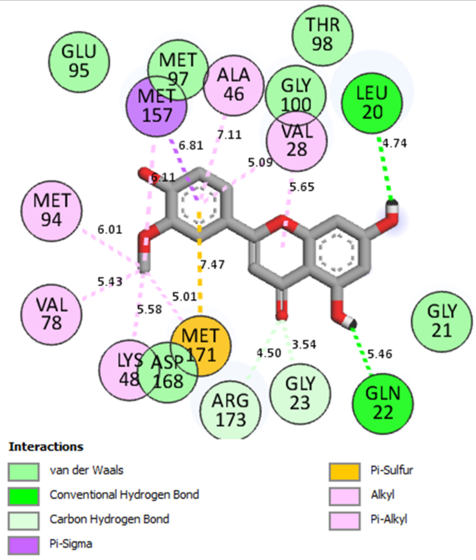

Isovitexin	60 ns	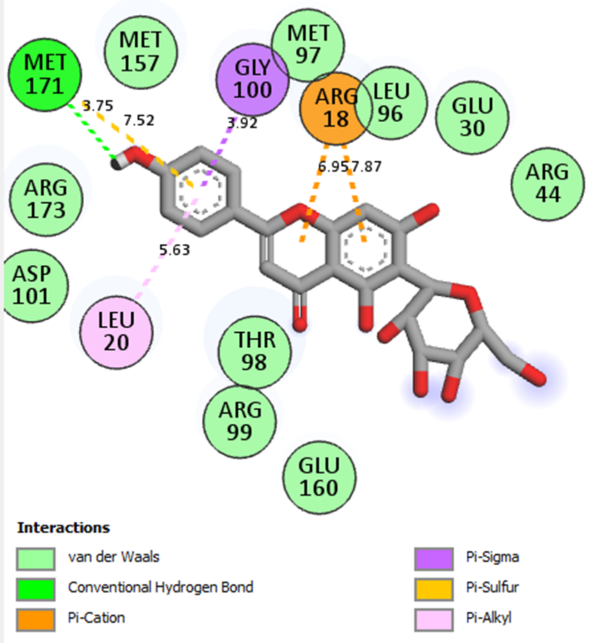
	30 ns	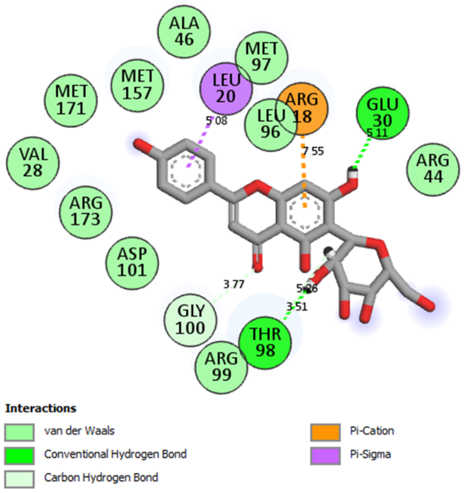
	0 ns	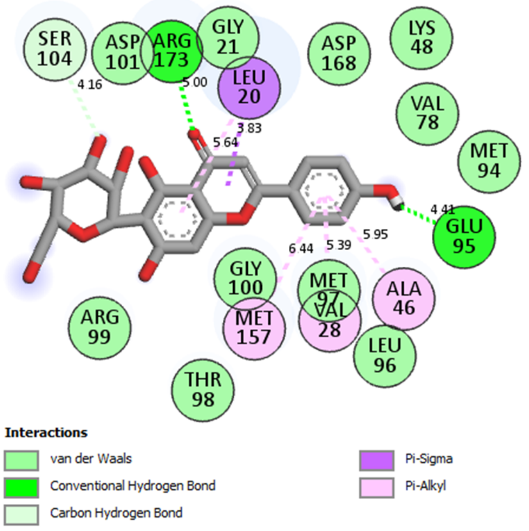

NVP-ADW742	60 ns	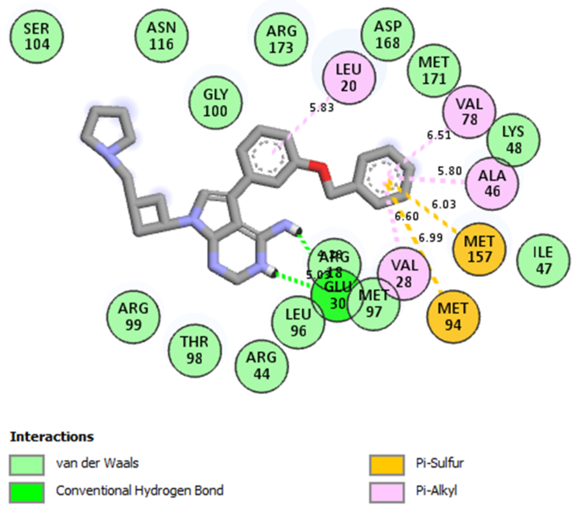
	30 ns	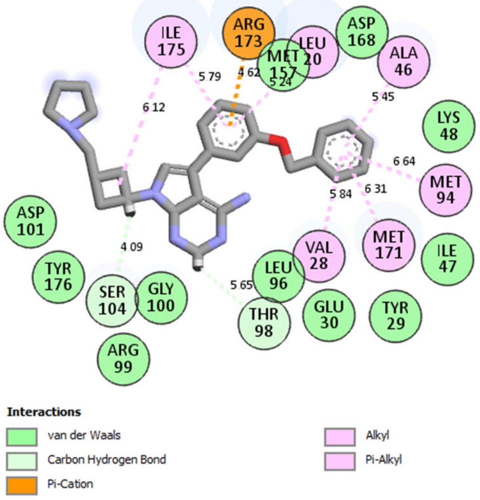
	0 ns	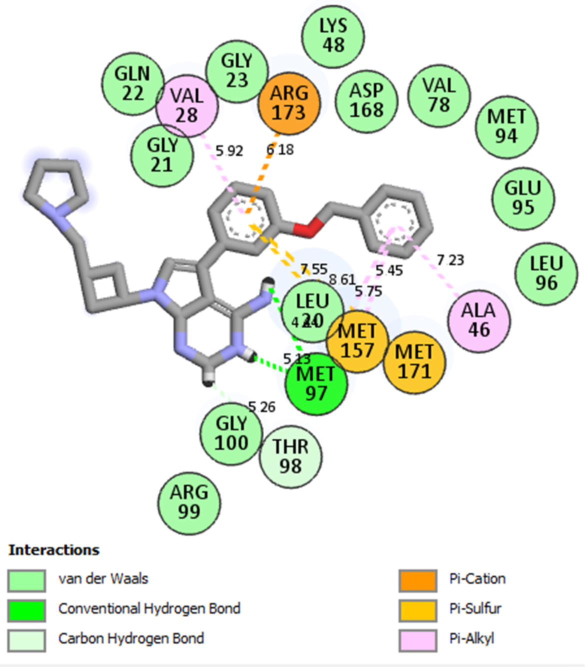

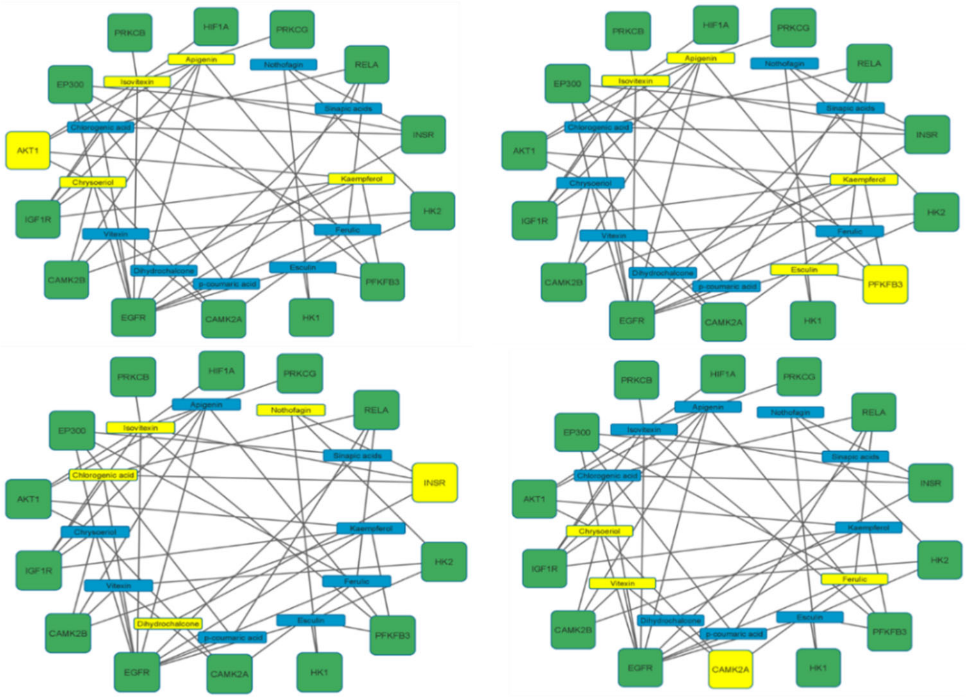

Figure S1. Rooibos compounds (4, 4, 4 and 3, respectively) with good interactions linked to other genes (AKT1, PFKFB3, INSR and CAMPK2A) related to the HIF-1 signaling pathway.
